# The Hybrid Type Polyketide Synthase SteelyA Is Required for cAMP Signalling in Early *Dictyostelium* Development

**DOI:** 10.1371/journal.pone.0106634

**Published:** 2014-09-15

**Authors:** Takaaki B. Narita, Zhi-hui Chen, Pauline Schaap, Tamao Saito

**Affiliations:** 1 Graduate School of Science and Technology, Sophia University, Tokyo, Japan; 2 Research Fellow of Japan Society for the Promotion of Science (DC2), Tokyo, Japan; 3 College of Life Sciences, University of Dundee, Dundee, United Kingdom; 4 Department of Materials and Life Sciences, Sophia University, Tokyo, Japan; MRC Laboratory of Molecular Biology, United Kingdom

## Abstract

**Background:**

In our previous study we found that the expression of *stlA* showed peaks both in the early and last stages of development and that a product of SteelyA, 4-methyl-5-pentylbenzene-1,3-diol (MPBD), controlled *Dictyostelium* spore maturation during the latter. In this study we focused on the role of SteelyA in early stage development.

**Principal Findings:**

Our *stlA* null mutant showed aggregation delay and abnormally small aggregation territories. Chemotaxis analysis revealed defective cAMP chemotaxis in the *stlA* null mutant. cAMP chemotaxis was restored by MPBD addition during early stage development. Assay for cAMP relay response revealed that the *stlA* null mutant had lower cAMP accumulation during aggregation, suggesting lower ACA activity than the wild type strain. Exogenous cAMP pulses rescued the aggregation defect of the *stlA* null strain in the absence of MPBD. Expression analysis of cAMP signalling genes revealed lower expression levels in the *stlA* null mutant during aggregation.

**Conclusion:**

Our data indicate a regulatory function by SteelyA on cAMP signalling during aggregation and show that SteelyA is indispensable for full activation of ACA.

## Introduction

*Dictyostelium* cells live in the soil as unicellular amoebae and consume bacteria and yeast as a food source. When their food source is depleted, *Dictyostelium* cells aggregate to form a multicellular organism. Cells in the aggregate start to differentiate into prestalk and prespore cells in a heterogeneous mixture which resembles salt and pepper [1]. Aggregates then transform into migrating slugs in which the differentiated cells sort to produce a prestalk-prespore pattern. After the slug stage, cells complete differentiation into their terminal types to form the stalk and spore cells of the mature fruiting body [1], [2]. Several small molecules are reported to control *Dictyostelium* development, amongst which the second messenger cAMP.

In *Dictyostelium*, cAMP acts not only as an intracellular second messenger but also as a secreted extracellular signal. During aggregation, secreted cAMP acts as a chemoattractant, which causes *Dictyostelium* cells to move together by chemotaxis [3]. In addition to inducing chemotaxis, the activation of cAMP receptor cAR1 also initiates a signal cascade that results in cAMP synthesis. Synthesized cAMP is then secreted to attract neighboring cells [4], [5]. cAMP also has an intracellular role in development, controlling many aspects of the developmental programme through the activation of PKA [6]–[10].

cAMP is produced by three distinct adenylyl cyclases during *Dictyostelium* development. These are ACA, ACB, and ACG. ACA produces the cAMP required for cell aggregation in early development [5], [11], ACB is involved in the maturation of stalk and spore cells [12], [13], and ACG controls prespore differentiation in the slug stage and also acts as an osmosensor that controls spore germination in the fruiting body [10], [13], [14].

The genome sequence of *Dictyostelium* was published in 2005 [15]. *Dictyostelium*, it turned out, has a massive potential for the production of polyketides, with 45 polyketide synthase (PKS) genes found in its genome [15], [16]. Two are novel types of hybrid PKSs called “Steely” [17], which to our best knowledge, are only found in the cellular slime molds. These novel enzymes are a fusion of iterative type I and type III PKSs. Iterative type I PKSs are large single polypeptides including multifunctional domains encoded by only a single gene and iteratively utilize some of their functional domains for a polyketide biosynthesis, whereas type III PKSs are single enzymes comprising of only *keto*-synthase domain [18], [19]. One of the Steely enzymes, SteelyB, was reported to produce Differentiation Inducing Factor-1 (DIF-1), an inducer of prestalk cell differentiation [17]. We showed earlier that a mutant that lacked the *stlB* gene made morphologically normal but structurally weak stalk and did not produce a basal disc or lower cup. These defects were recovered by the addition of DIF-1 in the medium, indicating that DIF-1 induces basal disc and lower cup formation among prestalk cells [17], [20]. DIF-1 has an additional role in developmental regulation [21], and both DIF-1 and the related molecule DIF-2 function as modulators of cAMP-induced chemotaxis [22]. This suggests that DIFs govern multiple aspects of the developmental programme.

Another Steely enzyme, SteelyA, was reported to make 4-methyl-5-pentylbenzene-1,3-diol (MPBD) *in vivo* and we showed that a *stlA* knockout mutant has defective spore maturation, which was recovered by the addition of MPBD to the mutant cells [23]. Previously, two distinct patterns of *stlA* gene expression were reported: one of them was that the gene was expressed during the early stage development [17], whereas another one was that the gene was expressed during only late stage [24]. We resolved these conflicting reports concerning the gene expression pattern, by re-examining the expression pattern with by RT-PCR using two different primer sets. It appeared that the expression of *stlA* peaked in the pre-aggregation stage, followed by smaller peak during the late culmination stage [23]. We also confirmed that the function of SteelyA during this second peak was the control of spore maturation [23], [25]. In subsequent research we specified the timing of MPBD-regulated spore maturation and found that MPBD was critical in the later stage of development [25].

In this study, we performed a detailed analysis of the function of SteelyA during early stage development and found that MPBD is required for normal expressions of cAMP signalling genes for aggregation.

## Materials and Methods

### Cell culture and development

The *Dictyostelium discoideum* Ax2 strain was grown in an axenic medium (HL-5) at 22°C [26]. The *stlA* null strain (Strain ID: DBS0236953) was grown in HL-5 medium supplemented with 10 µg/ml blasticidin S [17]. To initiate multicellular development, the axenically grown cells were harvested at a density of approximately 5 × 10^6^ cells/ml, washed twice in the phosphate buffer (PB) (2.7 mM Na_2_HPO_4_/10.7 mM K_2_HPO_4_, pH 6.2), and plated on PB agar plates (1.5% agar in PB) or nitrocellulose filters (0.45 µm pore size, Black gridded, 47 mm diameter, Millipore) at a density of 1×10^6^ cells/cm^2^. For gene expression analysis, developing cells were harvested every 3 hours until 12 h and used for RNA purification. For submerged streaming, the axenically grown cells were harvested and washed twice, and then submerged in 1 ml of PB at 5×10^5^ cells/cm^2^ in a culture dish and the depth of PB was about 3 mm.

### Chemotaxis assay and cAMP pulsing

Chemotaxis assay towards cAMP was performed as described [22] with a few modifications. Axenically grown cells in shaking culture were harvested in the log phase, washed in PB, and were starved at a density of 2×10^7^ cells/ml in PB in the presence or absence of MPBD for 6.5 h. Then, starved cells were washed twice in PB and resuspended in PB to a final density of 3×10^5^ cells/ml. Small portion of agar (∼10 g) was washed with water (500 ml each time) repeatedly and extensively. Then the agar was dried completely. The agar powder was dissolved in PB and boiled to prepare the hydrophobic agar plate. Three droplets of starved cells, 2 µl (3 mm diameter) each, were placed on 1% hydrophobic agar plate in the petri dish. Chemotaxis towards cAMP was tested by placing a 2 µl droplet of various concentrations of cAMP at 3 mm distance from the cell-filled droplet. The distribution of cells within their droplet was observed after 1 h, and droplets were scored ‘positive’ if at least twice as many cells had migrated to the side closer to the cAMP droplet than to the other side. The chemotaxis rate was calculated as the percentage of the number of ‘positive’ droplets to that of total droplets.

To find out the chemotactic response to folate, axenically grown cells in shaking culture were harvested in the log phase, washed twice in PB, and were resuspended in PB to a final density of 2×10^7^ cells/ml. Droplets of cells, 1 µl of the cell suspension, were placed on a hydrophobic agar plate at 3 mm distance from a 1 µl droplet of different concentrations of folate and were observed after 4 h from placing on the plate.

To examine the effect of cAMP pulsing on the *stlA* null strain, Ax2 or *stlA* null cells were harvested from the exponential growth phase and washed twice in PB. Washed cells were resuspended at a density of 2×10^7^ cells/ml in PB and then stimulated with 20 nM cAMP pulses every 6 min for 6 h. The pulsed cells were washed and then used for the chemotaxis assay or developed on nitrocellulose filters.

### Assay for cAMP relay response in intact cells

Cells were harvested in the log phase, washed twice, and distributed on non-nutrient agar plates (1.5% agar in 5 mM Na_2_HPO_4_•2H_2_O/5 mM KH_2_PO_4_ buffer, pH 6.5) at 1×10^8^ cells/plate. Cells were incubated for 16 h at 4°C and some hours at 22°C until aggregation territories started to form (2 h for the case of wild type cells and 3–4 h for the mutant cells) and then were harvested in the phosphate buffer (5 mM Na_2_HPO_4_•2H_2_O/5 mM KH_2_PO_4_, pH 6.5) and resuspended at a density of 1×10^8^ cells/ml. Cells were stimulated with 5 µM 2′-deoxycAMP in the presence of 5 mM DTT. Stimulation was terminated by adding an equal volume of 3.5% perchloric acid, and lysates were neutralized with 50% saturated KHCO_3_. cAMP levels were analyzed by isotope dilution assay, with [^3^H]cAMP as the competitor for binding to the bovine PKA regulatory subunit [27], [28].

### Gene expression analysis by qRT-PCR

Specific primer sets for qRT-PCR were designed to amplify a unique sequence located near the end of 3′ region of each gene (Table.1). BLAST similarity searches were used to confirm that each primer sequence amplified the unique sequence. Before using each primer set for qRT-PCR, genomic DNA was used as a template to evaluate the quality of primer set.

**Table 1:** Summary of the primer sets used in qRT-PCR.

Primer name	Sequence
*ig7* F	5′-TTACATTTATTAGACCCGAAACCAAGCG-3′
*ig7* R	5′-AACAGCTATCACCAAGCTTGATTAGCC-3′
*carA* F	5′-CCAGCACTCAATATTCTCC-3′
*carA* R	5′-ATGATGATAAAGAAGATGAAGATGAACC-3′
*acaA* F	5′-ATGCAATCCAATGCTCAAGATAATG-3′
*acaA* R	5′-AATGAGCCAATTTCACCCAAGAG-3′
*pdsA* F	5′-ATCCTCTGTCGCTTGTGATTGG-3′
*pdsA* R	5′-CCATTATTATTTGCTTCTTTTAATTGTTG-3′
*regA* F	5′-CCAAAACCACAAGAGCAAGAATCG-3′
*regA* R	5′-GAAGTTGAAGTTAAAGGAGCGGTCG-3′
*piaA* F	5′-GGTAATTTATCTTCACATATCACTG-3′
*piaA* R	5′-AATGAATCTTCTAGCACCTAAACG-3′
*dagA* F	5′-ACTACTAAAAGATCTCATCCAACTAC-3′
*dagA* R	5′-AAGCATCTTGAATGTATTGAGTCATTG-3′
*gpaB* F	5′-CAGCTTCAAAAACGACACG-3′
*gpaB* R	5′-CGAAAGAGCCTCAAACTATATCAAAG-3′
*csA* F	5′-CTGTTTCAGTTGGAGTTGGAGATG-3′
*csA* R	5′-CAGGTATCTTTTTAAACAATGCCC-3′

Total RNA was extracted from cells at the indicating time point using the RNeasy mini kit (Qiagen) according to the manufacturer's instructions and then the contaminating genomic DNA was eliminated using Recombinant DNase I (TaKaRa). DNase treatment was performed as follows: 50 µg total RNA was mixed with 10 units Recombinant DNase I and DNase I buffer (2 mM Tris-HCl; pH 7.5, 5 mM NaCl, 0.01 mM CaCl_2_, 5% glycerol) and incubated for 30 min at 37°C. The reaction was stopped by incubating with 25 mM EDTA at 80°C for 2 min. cDNA was synthesized by reverse transcription of 1 µg total RNA with oligo dT_15_ using ImProm-II Reverse Transcription System (Promega). qPCR was carried out with ABI PRISM 7000 Sequence Detection System (Applied Biosystems) and the amplifications were performed using iTaq Universal SYBR Green Supermix (Bio-Rad). The qPCR program was performed as follows: One cycle of 30 sec at 95°C followed by 40 cycles of 15 sec at 95°C, 1 min at 60°C for annealing, extension, and plate reading. Results of qRT-PCR were analyzed using the comparative C_T_ method [29] with the amplification of *ig7* (mitochondrial large rRNA) as a control.

## Results and Discussion

In previous studies we found that the expression profile of *stlA* peaks in the early stage of development and shows a weak peak in the last stage of development [23]. In this study we chose to understand SteelyA's function in the early stage of development by analyzing the *stlA* null mutant.

### StlA null strain shows small aggregation territories

**Figure 1:**
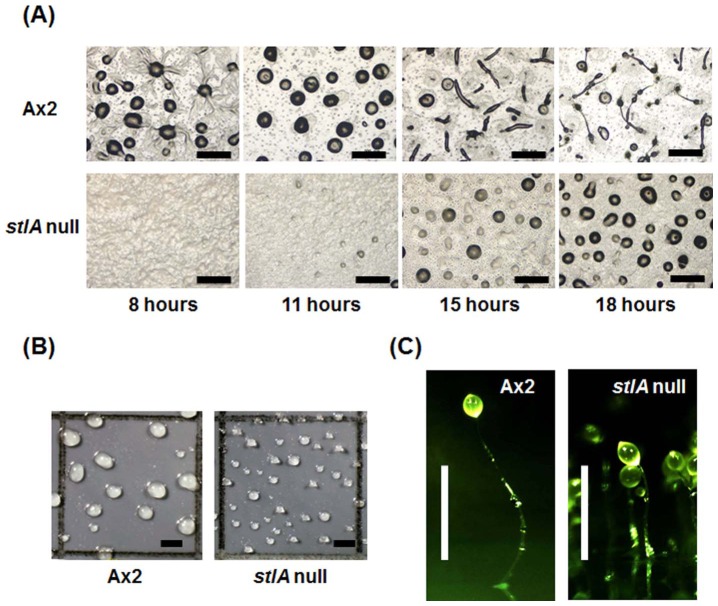
Developmental phenotype of *stlA* null mutant in the early stage. (A) Cells were developed on the PB agar plate at a density of 1×10^6^ cells/cm^2^. *StlA* null cells (bottom panels) showed aggregation delay compared to wild type strain, Ax2 cells (top panels). (B) Cells were developed on nitrocellulose filters at 1×10^6^ cells/cm^2^. The cells were observed when they finished forming multicellular aggregates. (C) Fruiting body of Ax2 and *stlA* null mutant developed on the PB agar plate at a density of 1×10^6^ cells/cm^2^. Bars indicate 500 µm (A and B) and 1 mm (C), respectively.

We first studied cell aggregation in the *stlA* null mutant and found that this process was delayed by about 3 h when compared with its parental strain Ax2. In some cases, the *stlA* null strain showed developmental delay by more than 7 h (Fig. 1A).

In addition to the aggregation delay, *stlA* null cells formed smaller aggregates than those of Ax2 cells. When cells were developed on a nitrocellulose filter, the *stlA* null cells made smaller and more numerous aggregates compared to Ax2 cells (Fig. 1B). As the result of forming small aggregates, fruiting bodies of the *stlA* null strain were smaller in size than those of the Ax2 strain (Fig. 1C).

**Figure 2:**
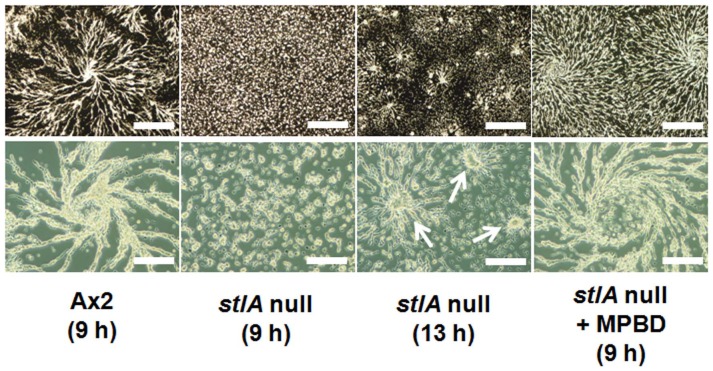
Aggregation stream formation under the submerged condition. Ax2 or *stlA* null cells were plated at 5×10^5^ cells/cm^2^ under the submersion. Photographs were taken at the indicated time. *StlA* null cells showed shorter aggregation streams and smaller aggregation territories than these of Ax2 cells (see arrows). In the presence of 200 nM MPBD, *stlA* null cells showed normal aggregation streams. These observations were confirmed in experiments repeated more than 3 independent times. Bars indicate 500 µm (top panels) and 200 µm (bottom panels).

To analyze the streaming behavior of *stlA* null cells in more detail, we observed aggregation under submerged conditions (Fig. 2). Under these conditions, *stlA* null cells made shorter aggregation streams and showed smaller aggregation territories than Ax2 cells. Since these aggregates were homogeneously distributed on the substratum the smaller aggregate sizes were not due to the uneven distribution of cells. To investigate whether these defects were due to the lack of MPBD, the major product of SteelyA enzyme, we added 200 nM MPBD directly to the mutant cells because 200 nM MPBD was needed to rescue the spore maturation defect in the mutant [23]. We found that the aggregation of *stlA* null cells was rescued in the presence of MPBD during starvation (Fig. 2). This observation suggests that MPBD is involved in cAMP-mediated aggregation.

### StlA null cells are impaired in chemotaxis towards cAMP

**Figure 3:**
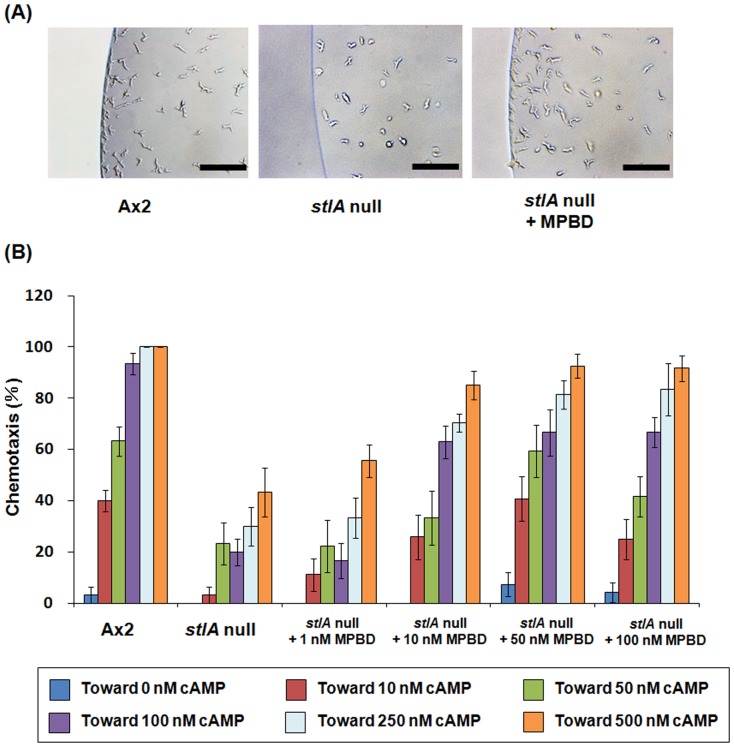
Chemotaxis defects in *stlA* null cells and the restoration by MPBD addition. (A) Cells were starved for 6.5 h in PB in the presence or absence of 200 nM MPBD and then the cell droplets were spotted next to 250 nM cAMP droplets on a hydrophobic agar plate. Cell droplets did not contain MPBD as starved cells were washed twice to eliminate MPBD before using this assay. The distribution of cells within droplets was observed and photographs were taken after 1 h. Bars: 100 µm. (B) Chemotaxis rate of the *stlA* null mutant with or without various doses of MPBD during starvation. The rate was calculated as the percentage of the number of ‘positive’ droplets to that of total droplets by the two droplet chemotaxis assay (see Materials and Methods). Cells were assayed for chemotaxis towards indicated concentrations of cAMP. Values are the means and SEM (bars) of 10 independent experiments (n = 10). The statistical analysis was performed by one-way ANOVA with Bonferroni's post-test. Significant differences (*p*<0.05) were shown in pairs as follows: Ax2–*stlA* null (towards 10, 50, 100, 250, and 500 nM cAMP), Ax2–*stlA* null with 1 nM MPBD (towards 100, 250, and 500 nM cAMP), Ax2–*stlA* null with 10 nM MPBD (towards 100 and 250 nM cAMP), *stlA* null–*stlA* null with 10 nM MPBD (towards 100, 250 and 500 nM cAMP), *stlA* null–*stlA* null with 50 nM MPBD (towards 100, 250 and 500 nM cAMP), *stlA* null–*stlA* null with 100 nM MPBD (towards 100, 250 and 500 nM cAMP), *stlA* null with 1 nM MPBD–*stlA* null with 10 nM MPBD (towards 100 nM cAMP), *stlA* null with 1 nM MPBD–*stlA* null with 50 nM MPBD (towards 100, 250 and 500 nM cAMP), and *stlA* null with 1 nM MPBD–*stlA* null with 100 nM MPBD (towards 100 and 500 nM cAMP).

**Figure 4:**
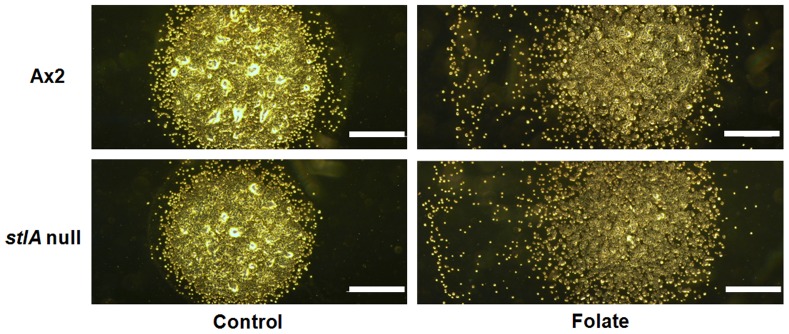
Representative result of chemotactic response to folate. Cells were harvested, washed twice, and resuspended in PB (2×10^7^ cells/ml). The cell droplet was spotted next to a droplet of 500 µM folate on a hydrophobic agar plate. Photographs were taken after 4 h from placing the cell droplet by phase- contrast microscopy. We took photographs of the cell droplet dividing into two pieces and then connected those photographs because we were not able to take it with one piece. Pictures presented are the connected photographs. Bars: 1 mm.

Small aggregation territories and delayed aggregation could result from defects in chemotaxis, cell adhesion or cAMP synthesis [30]–[32]. The observation that the *stlA* null mutant showed aggregation defect suggested that the mutant had a defect in chemotactic response to cAMP or cAMP production. To find out whether the aggregation defect was due to either chemotactic response to cAMP or cAMP production, we first investigated chemotaxis towards cAMP using the two droplet chemotaxis assay. As shown in Figure 3A, *stlA* null cells showed a chemotaxis defect towards 250 nM cAMP. In contrast, Ax2 cells showed clear cAMP chemotaxis under the same condition. This cAMP chemotaxis defect of the *stlA* null strain was recovered by addition of 200 nM MPBD in the buffer during 6.5 h starvation. This result indicates that the presence of MPBD during starvation induces competence for chemotaxis towards cAMP. We next examined the effect of various MPBD concentrations on chemotaxis. As shown in Figure 3B, MPBD improved cAMP chemotaxis in a dose dependent manner in the *stlA* null mutant. The minimal concentration for MPBD recovery of cAMP chemotaxis is about 100 nM and this concentration is similar to the recovery concentration of spore encapsulation in the *stlA* null mutant [23]. The defect in cAMP-induced chemotaxis was not due to the defect in cell motility because the *stlA* null strain showed the same chemotactic response to folate as the parent Ax2 (Fig. 4). Figure 4 shows a representative result of chemotactic response to 500 µM folate of each cell. We also examined the response to 250 µM or 100 µM folate and the same results were obtained (data not shown).

### cAMP-induced cAMP production is impaired in stlA null mutant

**Figure 5:**
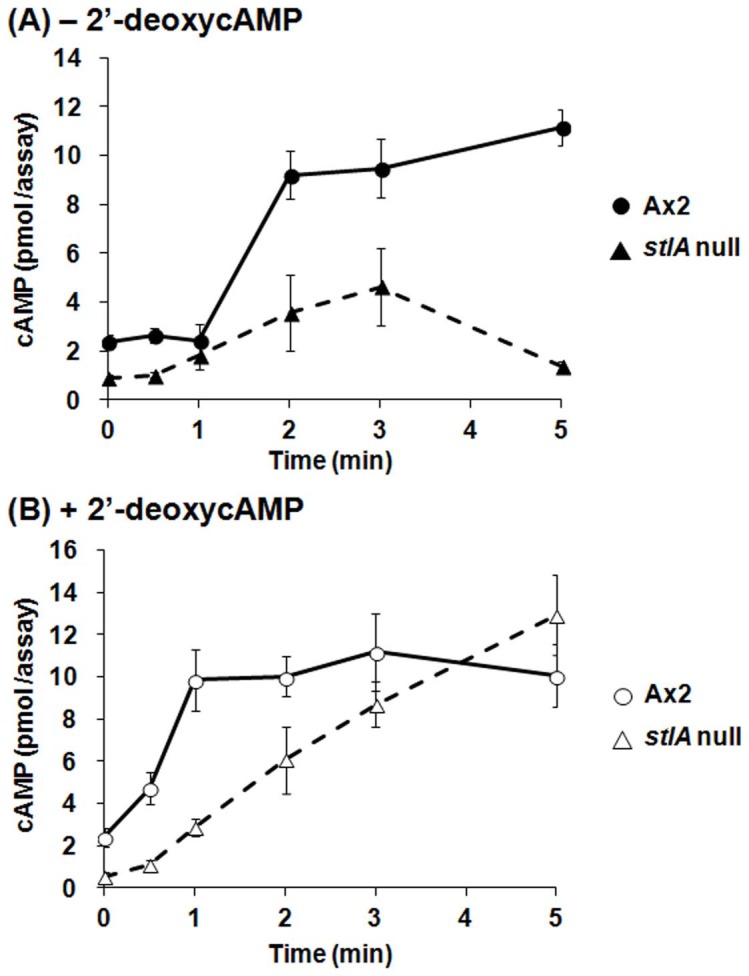
cAMP relay response in *stlA* null mutant. Cells were developed on non-nutrient agar plates until aggregation territories started to form. Cells were resuspended in PB and stimulated with 5 µM 2′-deoxycAMP and 5 mM DTT or with 5 mM DTT only. (A) cAMP accumulation in Ax2 or *stlA* null cells without 2′-deoxycAMP stimulation. The cAMP accumulation in *stlA* null cells was less than half of that in Ax2 cells. (B) cAMP accumulation in each cell that ACA was activated by 2′-deoxycAMP stimulation. *StlA* null cells showed a slower production rate than Ax2 cells. However, the accumulation in *stlA* null cells was almost the same as that in Ax2 cells eventually. Values are the means and SD (bars) of 3 independent experiments (n = 3).

Next, we focused on cAMP-induced cAMP production in the *stlA* null mutant. When cells were stimulated by extracellular cAMP during the aggregation stage, they soon started to produce extracellular cAMP to transmit the aggregation signal to adjacent cells. Figure 5 shows the results of cAMP relay response assay. When cells were stimulated with cAMP analogue, 2′-deoxy cAMP, cAMP receptor was stimulated and then ACA was activated. We measured the cAMP accumulation in the 2′-deoxy cAMP stimulated cells that ACA was activated. Figure 5 shows the result with 2′-deoxy cAMP stimulation. Without 2′-deoxy cAMP stimulation, *stlA* null cells accumulated less than half of cAMP in Ax2 cells (Fig. 5A). On the other hand, with 2′-deoxy cAMP stimulation, *stlA* null cells eventually showed almost the same cAMP accumulation, but had slower production kinetics than Ax2 cells (Fig. 5B).

**Figure 6:**
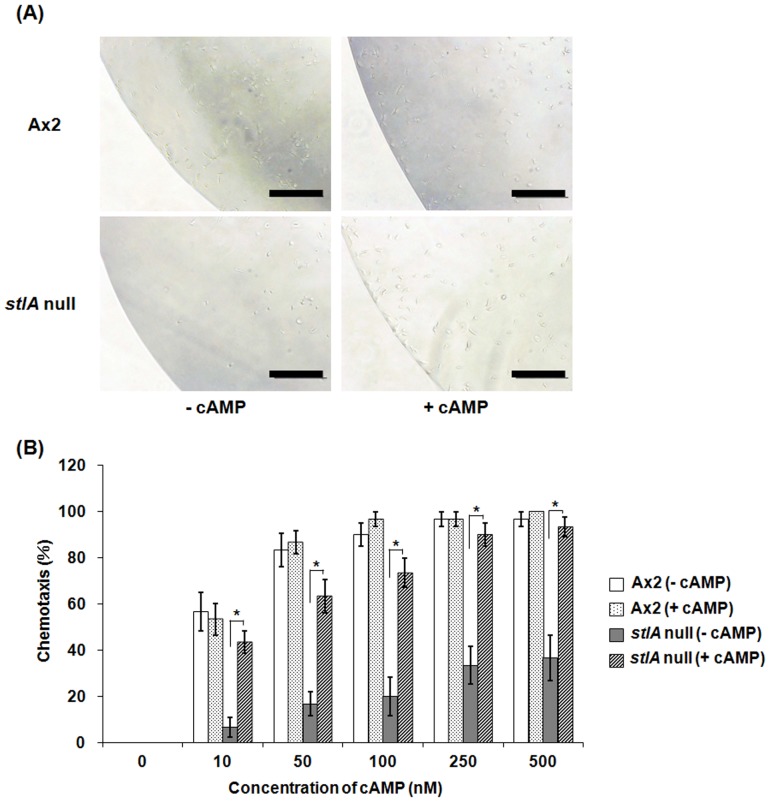
Effect of exogenous cAMP pulses on chemotaxis in *stlA* null cells. (A) Cells were starved at 2×10^7^ cells/ml in PB with or without 20 nM cAMP pulses every 6 min for 6 h. Droplets of pulsed cells were spotted next to 500 nM cAMP droplets on hydrophobic agar plates. Photographs were taken after 1 h. Bars: 200 µm. (B) Chemotaxis rate of the *stlA* null mutant with or without cAMP pulsing. Cells were assayed for chemotaxis towards indicated concentrations of cAMP using the two droplet chemotaxis assay and their chemotaxis rates were calculated (see Materials and Methods). Values are the means and SEM (bars) of 10 independent experiments (n = 10). *p<0.001 (two paired t-test).

**Figure 7:**
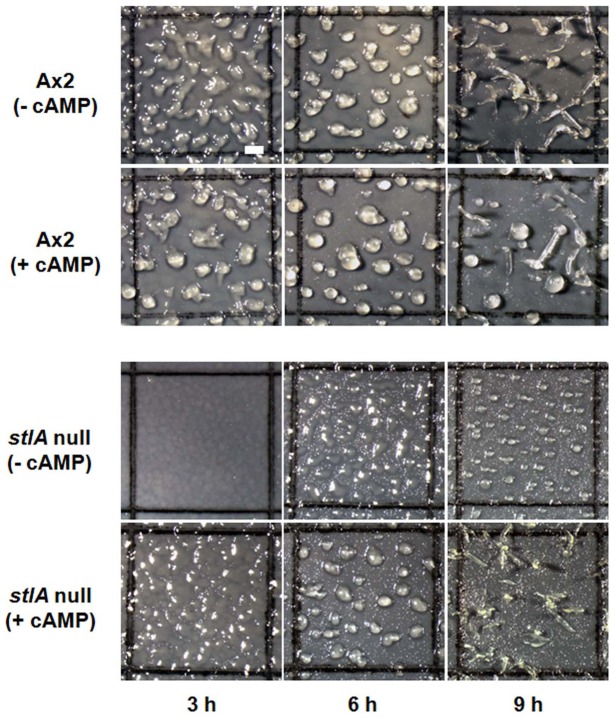
cAMP pulsed *stlA* null cells show normal aggregation in the absence of MPBD. Cells were stimulated by 20 nM cAMP pulses every 6 min for 6 h and then were developed on nitrocellulose filters at 1×10^6^ cells/cm^2^. Time points on panels indicate time elapsed since cells were washed following the completion of cAMP pulsing. cAMP pulsed *stlA* null cells show normal development without developmental delay. We observed the same phenomena in experiments repeated 3 independent times. Bar: 400 µm.

These results strongly suggested that the *stlA* null mutant had a defect in the cAMP signal relay, especially related to the production of cAMP in the early aggregation stage. To confirm this, the cells were starved and stimulated with periodical addition of cAMP. Figure 6A shows the rescue of cAMP chemotaxis in *stlA* null cells by cAMP pulsing. When *stlA* null cells were stimulated with periodical cAMP addition during starvation, they showed the normal cAMP chemotaxis. This recovery of cAMP chemotaxis by cAMP pulsing indicates that *stlA* null cells have a defect in periodical cAMP production. Figure 6B indicates the dose dependency of cAMP chemotaxis in cAMP pulsed cells examined by the two droplet chemotaxis assay. Also, cAMP pulsed *stlA* null cells showed normal development without aggregation delay, that is, the aggregation defect of the mutant was recovered by cAMP pulsing in the absence of MPBD (Fig. 7). These results suggested that MPBD was involved in cAMP signal relay and regulates the synthesis and/or release of cAMP in the early stage of development.

### cAMP signalling genes are down regulated in stlA null mutant

The observation that the *stlA* null mutant responds chemotactically to folate but not to cAMP suggests a defect in cAMP signal transduction. cAMP-induced chemotaxis is mediated by complex parallel signal transduction cascade. The observation that pretreatment with MPBD restores cAMP-induced chemotaxis in the *stlA* null mutant suggests that MPBD may induce the expression of genes required for this response.

**Figure 8:**
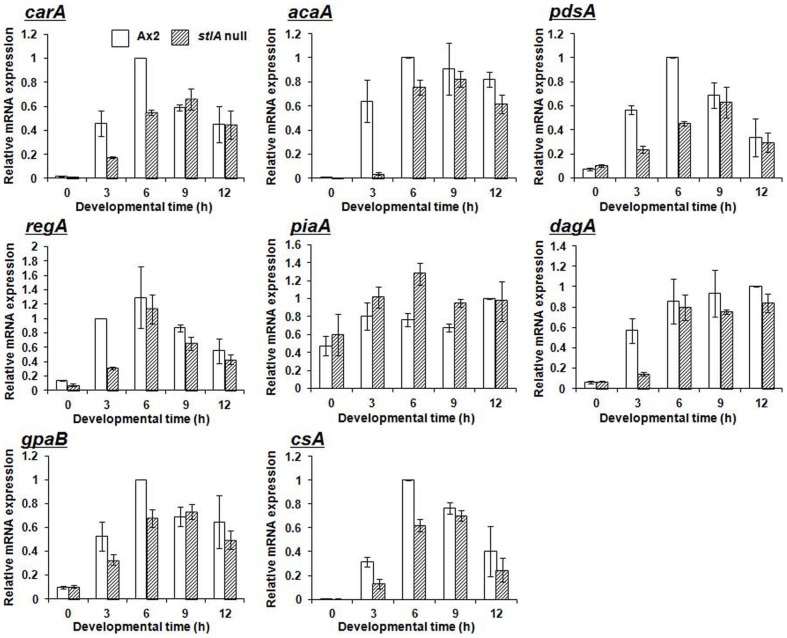
Expression patterns of cAMP signalling genes in *stlA* null mutant. Expression levels of each gene were analyzed by qRT-PCR. The levels were normalized to that of the endogenous control *ig7* (mitochondrial large rRNA). In addition, the expression value of Ax2 at some time point (*carA*, *acaA*, *pdsA*, *gpaB* and *csA*: 6 h, *regA*: 3 h, *piaA* and *dagA*: 12 h) in each gene was used as an internal standard and values were normalized it. Values indicated in graphs are the means and SD (bar) of 3 independent experiments, which were performed by using independent total RNA.

To determine which step(s) caused the defective chemotaxis in the *stlA* null mutant we examined the expression pattern of cAMP signalling genes in *stlA* null and Ax2 cells. Figure 8 shows the gene expression patterns of key genes in cAMP chemotaxis by quantitative RT-PCR. We analyzed the genes coding for the cAMP receptor (*carA*), adenylyl cyclase (*acaA*), secreted cAMP phosphodiesterase (*pdsA*) [33], intracellular cAMP phosphodiesterase (*regA*) [34], positive regulators of adenylyl cyclase (*piaA* and *dagA*) [35], [36] and an *α* subunit of G-protein coupled with a cAMP receptor (*gpaB*) [37]. The mRNA levels of these genes were lower in the mutant cells than in Ax2 cells, with the sole exception of the *piaA* gene. The reason for this difference in *piaA* gene expression from other cAMP signalling genes will be discussed later.

This decreased expression pattern was especially clear at 3 h when aggregation genes started to express. Although the maximal mRNA levels of these genes were initially lower in the *stlA* null mutant than in the Ax2, their levels at 9 h were almost the same as those of the Ax2 cells (9 h in Figure 8). At this point (9 h), the Ax2 cells had reached the late aggregation stage, while *stlA* null cells were early aggregation. We also examined the expression levels of a gene coding for cell-cell adhesion protein (*csA*) [38]. This gene is strictly developmentally regulated and cAMP inducible [39]. Although the expression of *csA* was lowered and delayed in the *stlA* null mutant compared to Ax2, its mRNA level in the *stlA* null mutant was the same as Ax2 by 9 h. These results indicate that at least at 9 h after starvation (t9), the expression levels of cAMP signalling genes in the *stlA* null mutant are enough to induce the expression of cAMP inducible genes, and as a result, *csA* is expressed normally. We also confirmed that MPBD rescued *carA* and *acaA* expression defects in the *stlA* null mutant (data not shown).

As mentioned above, the expression of *piaA*, a positive regulator of adenylyl cyclase, was not down regulated as other genes in the *stlA* null mutant but rather up regulated. This regulation of *piaA* may be due to the difference of pathways activating ACA between CRAC (Cytosolic Regulator of Adenylyl Cyclase) and Pia (coded by *dagA* and *piaA*, respectively). Pia is a component of the target of rapamycin complex 2 (TORC2) [40]. TORC2 plays roles in activation of ACA and PKB [40]–[43]. By reconstitution experiments using cells lacking both CRAC and Pia, it has shown that both CRAC and Pia are absolutely required for ACA activity [35]. However, it has also reported that CRAC is not required for TORC2 formation [40]. In our *stlA* null mutant, *dagA* was down regulated but expressed partially. This means that the CRAC pathway in the *stlA* null mutant is partially active but has only a small effect on ACA. We propose that the *stlA* null mutant should make use of the TORC2 pathway to compensate for reduced ACA activity caused by down regulated *dagA*. As a result, the expression of *piaA* may be up regulated in the *stlA* null mutant.

From above data, we concluded that the function of SteelyA during early stage is to make starving cells aggregation competent by regulating the cAMP signalling gene expression. This means both the chemotactic response to cAMP and adenylyl cyclase activity required for aggregation are controlled by MPBD, a major product of SteelyA.

In this study, we focused on the role of SteelyA during the early stage of development and showed that the polyketide MPBD produced by SteelyA is required for the expression of cAMP signalling genes during early development. A *stlA* null mutant had aggregation defects because their expression in the mutant was down regulated.

The *stlA* null mutant showed similar phenotype to the *padA* ^−^ mutant [30], [44]. In both mutant, chemotactic cAMP response was impaired and cAMP signalling genes were down regulated. However, the aggregation defect in the *padA* ^−^ mutant is not rescued by cAMP pulses because cAMP pulses are not able to fully induce cAMP relay genes in the mutant. In addition, accumulated extracellular cAMP levels in the *padA* ^−^ mutant are almost the same as that in wild type unlike the *stlA* null mutant [30]. In contrast, the aggregation defect in the *stlA* null mutant was restored by cAMP pulses and the mutant showed lower accumulated cAMP levels. MPBD signalling cascade for induction of cAMP signalling genes, therefore, is probably different pathway from PadA signalling cascade. MPBD may act on the same pathway as cAMP pulses for stimulation of cAMP signalling gene expression because cAMP pulsed *stlA* null cells show normal aggregation in the absence of MPBD.

MPBD acts as a spore maturation factor during the late stage of development [23], [25]. Anjard et al. reported that MPBD might bind to CrlA, the GPCR coupled to G*α*1, and inhibit GskA to induce spore differentiation by SDF-1 [45]. CrlA, suggested as an MPBD receptor, is also expressed during early stage [46], [47]. However, the *crlA* knockout mutant was reported as forming larger aggregates than wild type cells and exhibited normal chemotaxis to cAMP [46]. Although there is the difference of parental strains between *crlA* and *stlA* mutant (KAx3 and Ax2, respectively), the phenotype of *crlA* mutant differs from that of *stlA* null mutant. Therefore, MPBD–CrlA pathway might be not used to modulate the expression of cAMP signalling genes during the early stage of development. MPBD might regulate those expressions by acting through some receptor other than CrlA.
